# The ET-1-mediated carbonylation and degradation of ANXA1 induce inflammatory phenotype and proliferation of pulmonary artery smooth muscle cells in HPS

**DOI:** 10.1371/journal.pone.0175443

**Published:** 2017-04-17

**Authors:** Jing He, Bin Yi, Yang Chen, Qing Huang, Huan Wang, Kaizhi Lu, Weiling Fu

**Affiliations:** 1 Department of Anesthesia, Southwest Hospital, The Third Military Medical University, Chongqing, China; 2 Department of Laboratory Medicine, Southwest Hospital, Third Military Medical University, Chongqing, China; Augusta University, UNITED STATES

## Abstract

Hepatopulmonary syndrome (HPS) is a serious complication of advanced liver disease, which markedly increases mortality. Pulmonary vascular remodelling (PVR) induced by circulating mediators plays an important role in the pathogenesis of HPS, while the underlying mechanism remains undefined. In the present study, we reported that endothelin-1 (ET-1) is up-regulated and annexin A1(ANXA1) is down-regulated in HPS rat, and ET-1 decreases the ANXA1 expression in a dose-dependent manner in rat pulmonary arterial smooth muscle cells (PASMCs). Then, we showed that ANXA1 can decrease nuclear p-ERK1/2 accumulation and decrease the cyclin D1 expression, thus resulting in the subsequent inhibition of PASMCs proliferation. As previously reported, we confirmed that ET-1 decreases the ANXA1 protein levels by the carbonylation and degradation of ANXA1. In conclusion, our research links the signaling cascade of ET1-ANXA1-cell proliferation to a potential therapeutic strategy for blocking IPS-associated PVR.

## Introduction

Liver plays an important role in homoeostasis, and chronic liver diseases, such as cirrhosis and portal hypertension, often lead to the alterations of vasculature in multiple organs[1]. Hepatopulmonary syndrome(HPS), an advanced liver disease causing lung vascular disorder, has drawn more and more attention in recent years because of it high mortality. HPS has three clinical characteristics: advanced chronic liver disease (CLD), intrapulmonary vasodilatation (IPVD), and arterial hypoxemia[2]. Currently, orthotopic liver transplantation(OLT) is the only viable treatment option. As the pathophysiological mechanisms associated with HPS have not been fully identified, there is no other effective treatment strategies for HPS[3, 4].

As one of the major components of blood vessel wall, pulmonary artery smooth muscle cells (PASMCs) play an important role in maintaining vascular structure and functions. In the arterial wall, PASMCs are normally quiescent and seldom to proliferate (<0.05%), and mainly in the G0/G1 phases of the cell cycle[5]. However, cytokines and growth factors during HPS cause PASMCs to re-enter cell cycle from the quiescence to proliferation state, which plays a key role in the formation and progression of PVR associated with HPS[1, 6].

ET-1 is a vasoactive substance that potently triggers constriction of vessels and thus plays an essential role in guarding the integrity of the vasculature[7]. However, elevated ET-1 levels can activate vascular smooth muscle cells, inducing proliferation, hypertrophy, and synthesis of extracellular matrix, which lead to a diverse range of cardiovascular diseases including atherosclerosis, cardiac hypertrophy and pulmonary hypertension[8, 9]. The detrimental effects exerted by ET-1 on the vasculature include excessive accumulation of reactive oxygen species[10], promotion of vascular smooth muscle cells proliferation[11], and chronic vascular inflammation[12].

ANXA1 is a calcium- and phospholipid-binding protein involved in various biological processes, including differentiation, proliferation, apoptosis and migration[13]. Altered expression profiles, subcellularlocalization, phosphorylation, carbonylation, and/or specific modulation of mitogenic signals are all possible mechanisms by which ANXA1 protein could be changed to achieve its different biological effects[14]. Suzuki and co-workers[15] previously reported that ANXA1 regulates the proliferation of PASMCs. However, whether ANXA1 is involved in the regulation of PASMCs proliferation in HPS has not been defined.

In this study, we investigated the role of ANXA1 in PASMC proliferation in relation to HPS. Our studies demonstrated that the ET-1-mediated carbonylation and degradation of ANXA1 promote PASMCs in HPS.

## Materials and methods

### Reagents and antibodies

ET-1 was purchased from Peninsula (San Carlos,CA). Real-time PCR kits were obtained from Qiagen (Valencia, CA). The anti-DNP antibody were obtained from Dako (Cambs, UK); mouse anti-rat preproET-1 antibody; rabbit anti-rat phospho-ERK1/2 antibody, rabbit anti-rat cyclin D1 antibody, rabbit anti-rat ANXA1 antibody, rabbit anti-rat-actin antibody, HRP-conjugated goat anti-rabbit IgG and HRP-conjugated goat anti-mouse IgG were provided by Abcam (Cambridge, USA). The SDS-PAGE gel kit, the mammalian protein extraction kit, the BCA protein assay kit and the eECL western blot kit was purchased from CWBIO (Beijing, China). MG132 was purchased from the Haoran Biological Technology Company (Shanghai, China). Entranster TM-R was obtained from Engreen Biosystem (Beijing,China), CCK-8 was provided by Pierce (Rockford IL, USA). ^3^H-TdR Kindly provided was kindly provided by the Department of Nuclear Medicine(Southwest Hospital, China).ROS Assay Kit was purchased from Jiancheng Bioengineering Institute(Nanjing, China). ELISA assay kits were purchased from R&D Systems (Minneapolis, USA). Rat Endothelin 1 (ET-1) ELISA Kit were obtained from Abcam (Cambridge, USA).

### HPS rat model

All procedures performed on the rats were conducted according to the guidelines from the National Institutes of Health. The study’s protocol was approved by the committee on Animal Research of Southwest Hospital of the Third Military Medical University. The HPS rat model was established as described in our previous study[16]. Briefly, Sprague-Dawley rats (180–220 g) were chosen to induce HPS by common bile duct ligation (CBDL). The CBDL operation was performed after anesthetizing the rats with 2% isoflurane delivered in oxygen. The common bile duct was exposed and doubly ligated with 3–0 silk. Those animals receiving analgesia (0.1 mg/kg buprenorphine, SC, q12h) post-operatively; the condition of the animals was monitored daily. HPS group rats obtained from these CBDL rats meeting the following criteria: 1) gas exchange abnormalities (PaO_2_<85 mmHg and P(A-a)O_2_>18 mmHg); 2) intrapulmonary vascular dilation, confirmed by histopathological analysis. Control group rats were obtained from the sham-operated rats. No animal became severely ill or died at any time prior to the experiment endpoint. Clinical signs used to determine when to euthanize the animals include: quickly losing weight, too weak to eat and drink, infection of organs which have no response to drug therapy.

### Cell culture

The rat PASMCs were isolated from rat pulmonary arteries as previously described. Briefly, pulmonary artery was harvested under sterile conditions. After the internal and external membrane were removed, the middle membranes were cut into 1 mm ×1mm size pieces, and were cultured by tissue-piece inoculation method, added to DMEM medium with low concentration of glucose(DMEM/F121:1). The smooth muscle cells were identified by immunohistochemistry staining with the SM-α-actin and calponin,and the purity of the third passage PASMCs was more than 98%. The PASMCs were used for experiments between passages 4 and 8.

### Transfection

After growth arrest, PASMCs were transfected with different groups of transfection complexes. Transfection complexes were prepared according to the manufacturer's instructions. Ad-ANXA1 were used to overexpress ANXA1 in PASMCs. At 24 h prior to treatment, Ad-ANXA1, at a multiplicity of infection (MOI) of 60, was added into the medium of the Ad-ANXA1 transfection group. The flask of this group was placed upside-down on an inverted immunofluorescence microscope to observe the transfection efficiency.

### Western blotting and immunoprecipitation

After cultured cells were lysed and subjected to total and nuclear protein extraction, equal quantities of protein were analysed by sodium dodecyl sulphate polyacrylamide gel electrophoresis (SDS-PAGE) and then transferred onto the PVDF membranes. The membranes were blocked for 1 h using the blocking solution and then incubated overnight with the primary antibody (1:1000 dilution) at 4°C. After washing the membrane three times with Tris-Buffered Saline Tween-20 (TBST), the membrane was incubated with HRP-conjugated rabbit anti-goat IgG or HRP-conjugated goat anti-mouse IgG at a 1:1000 dilution for 1 h at room temperature before being washed again in TBST and visualised with an enhanced chemiluminescence. Finally, the immunoreactive bands were scanned and stored using a gel imaging system. The optical density of the immunoreactivity was measured and analysed with an Alpha Imager.

For immunoprecipitation, protein lysates were diluted to a concentration of 2 mg/mL and immunoprecipitated for ANXA1 for 4 h at 4°C. 20μl of 50% v/v protein G Sepharose was then added, and the mixture was incubated for 4 h at 4°C, followed by centrifugation to recover the protein G Sepharose pellets. After three washes in lysis buffer, the immunocomplexes were released by addition of SDS sample buffer. ANXA1 carbonylation was detected by western blot using an anti-DNP antibody.

### PCR

Total RNA was extracted using Tripure Isolation Reagent. For the measurement of ANXA1 mRNA expression, RNA (2μg) was reverse-transcribed using the Real-time RT-PCR assay kits, followed by real-time PCR using the Fast-Plus EvaGreen master mix. The amplification and detection of specific products were performed using the iCycleriQ Real-Time PCR Detection System. Glyceraldehyde 3-phosphate dehydrogenase (GADPH) was used for other template normalisations.

### Immunohistochemistry

The lung tissues were inflated with 0.75 ml of 10% formalin overnight and transferred to 70% ethanol, embedded in paraffin, and sectioned into 5 mm slices. The sections were subjected to antigen retrieval, blocked, incubated with an anti-ANXA1 or anti-preproET-1 antibody(1:200) for 2 h at 37°C, and incubated with a horseradish peroxidase (HRP)-conjugated goat anti-rabbit secondary antibody (1:200) for 90 min at room temperature. Each lung section was evaluated via confocal microscopy (Zeiss LSM-510). The immunostained sections were quantitatively characterized via a digital image analysis using Image Pro-Plus, version 6.0.

### Enzyme-linked immunosorbent assay

The concentrations of IL-1β, IL-6 and TNF-α in medium and ET-1 in lung tissue were examined by ELISA assay kits following manufacturer’s instructions (R&D Systems, USA). PASMCs cells were seeded in 96-well plates at 4 × 104 cells/well for 24 hours before the experiments. After treatment, the medium was collected after centrifugation at 1000g for 20min and 100μl of supernatant was used for detection. The absorbance was read using a spectrophotometer at a wavelength of 450nm. The concentrations were calculated according to the standard curve and presented as pg/ml.

### Thymidine incorporation assay

The same number of PASMCs was seeded into a 96-well plate in complete medium. After 24 h of synchronous growth, cells were transfected with ANXA1 or adenovirus empty vector or vehicle according to the manufacturer’s instructions. Next, the cells were incubated with DMEM containing ET-1 or vehicle for the indicated period and supplemented with 1Ci ^3^H-TdR during the last 6h of the treatment period. The incorporation was stopped with cold PBS solution, and 0.25% trypsin was added to digest the cells, separating them from the cell wall. The cells were collected on a glass fibre filter with a multi-head harvester. The cells were washed with physiological saline 3 times, stabilised with 10% trichloroacetic acid, decolourised with absolute ethanol, dried for 30 min at 80°C, transferred into scintillation fluid and counted with a liquid scintillation counter (counts/min, cpm).

### CCK-8 assay

Cellular proliferation was detected by the Cell Counting Kit-8 (CCK-8) assay. Accordingly, 24 h after the same number of PASMCs were seeded. After transfection, the cells were incubated with DMEM containing ET-1 or vehicle for the indicated time periods. At the end of the treatment, 10μl CCK-8 solution was added to each well, and the cells were cultured for 2 h more. After that, viable cells were detected by measuring the absorbance value at 450 nm using a microplate absorbance reader.

### Detection of reactive oxygen species

Reactive oxygen species in hippocampus were detected using a ROS Assay Kit (Nanjing Jiancheng Bioengineering Institute, China) following the manufacturer’s instructions.

### Statistical analysis

All data were expressed as the means±SEM. Comparisons between groups were carried out using Student's t-test or a Mann-Whitney U-test for parametric or non-parametric data, respectively. The results were considered to be statistically significant when p< 0.05.

## Results

### Pulmonary vascular remodeling and distribution of ET-1 is dramatically changed in HPS rat lung

**Fig 1:**
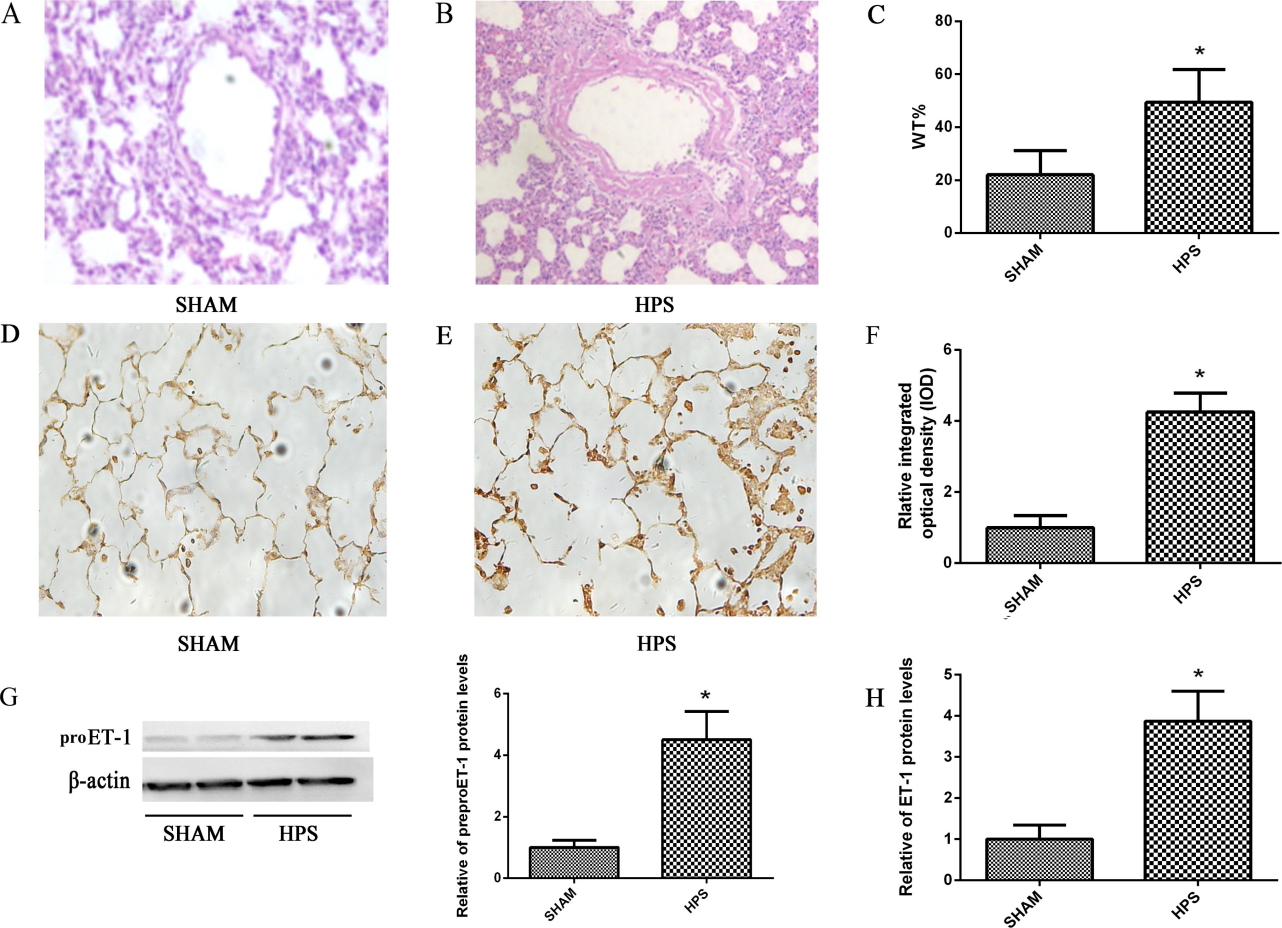
Pulmonary vascular remodeling and distribution of ET-1 in HPS rat lung. (A.B) Representative images of pulmonary vascular remodeling sections from HPS and SHAM group rats stained with HE. (C) The arteriole wall thickness/vascular extemal diameter (WT%) is displayed. (D.E) Immunohistochemical localization of preproET-1 in lung sections from normal and HPS rats. (F) The relative level of preproET-1 protein expression compared with SHAM group is displayed. (G) Western blotting and graphical summaries of preproET-1 protein levels in lung tissues of HPS rats. β-actin was used as an internal control. (H) Expression of ET-1 was measured in the lung tissue from HPS and SHAM group rats by ELISA. All datas were representative of four independent experiments. Values were expressed as means±SEM. *P<0.05 compared with SHAM group.

Histological analysis showed that medial thickness and collagen deposition of pulmonary intra-alveolar arteries were significantly increased in HPS rat (Fig 1A, 1B and 1C). As ET-1 plays an improtant role in PVR, we measured pulmonary levels of preproET-1 (an precursor of endothelin-1) in HPS and SHAM group rat by immunoblot analysis. Pulmonary preproET-1 levels in HPS group were 4.6-fold higher than Shamed group after 3 weeks(Fig 1G). To assess the distribution of preproET-1 in lung, we performed immunohistochemical staining. In SHAM group, preproET-1 was dominantly located in the pulmonary microvascular regions, with minimal staining in alveolar epithelial cells (Fig 1D), There was a substantial level increase of preproET-1 immunohistochemical staining in HPS group. The distribution of preproET-1 staining was detected in much more regions, including pulmonary arterial smooth muscle cells (PASMCs), pulmonary microvascular endothelial cells(PMVECs), alveolar epithelial cells and macrophages(Fig 1E). The relative integrated optical density (IOD) in HPS group was 4.3-fold higher than SHAM group after 3 weeks(Fig 1F). Rat ET-1 ELISA Kit were used to directly measure the expression of ET-1 in lung tissue, and ET-1 levels in HPS group were 3.8-fold higher than SHAM group after 3 weeks(Fig 1H).

### Pulmonary vascular remodeling of HPS is highly correlated with the ANXA1 inhibition by ET-1

**Fig 2:**
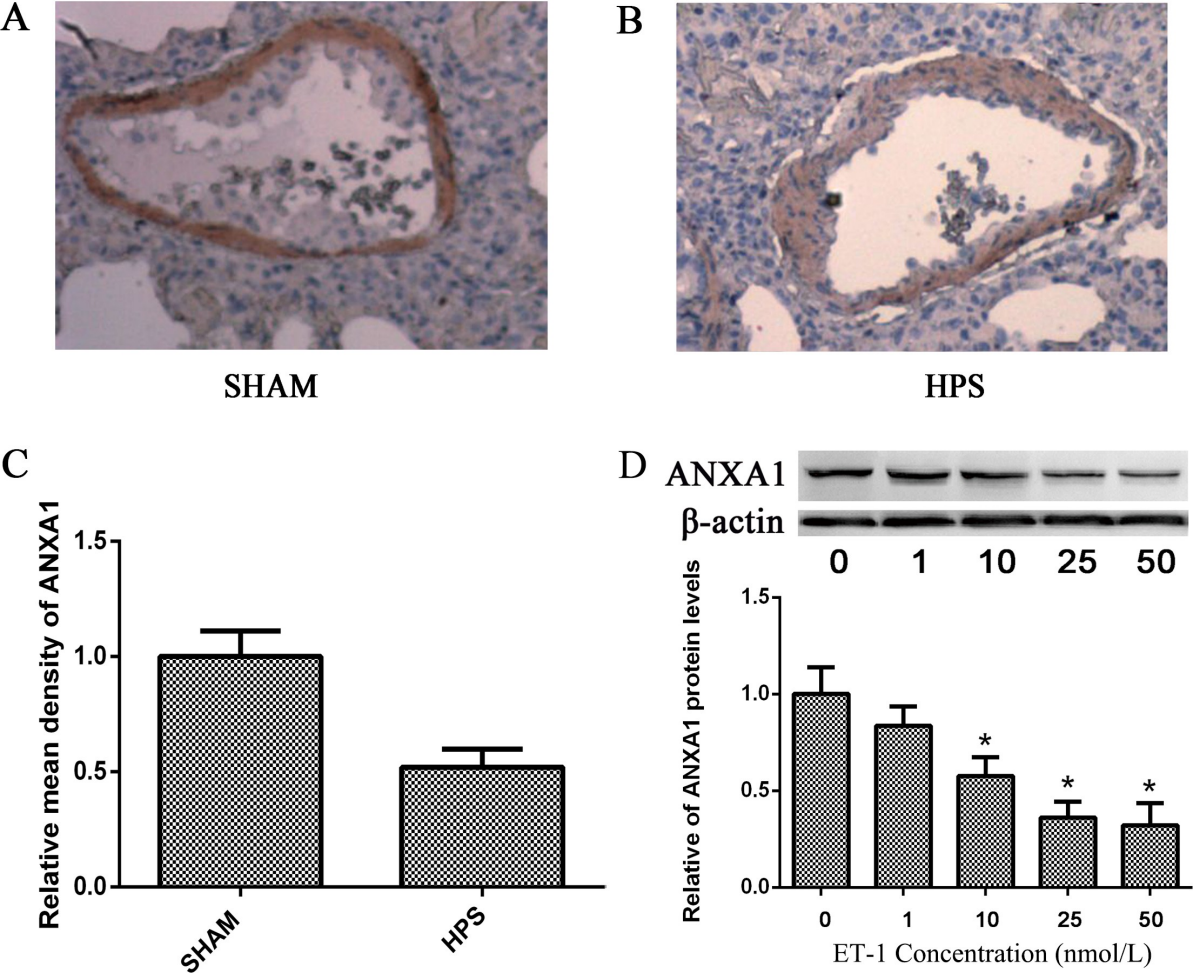
Pulmonary vascular remodeling of HPS was highly correlated with the ANXA1 inhibition by ET-1. (A.B) Representative images of ANXA1 protein expression in lung tissues using immunohistochemistry. (C) The relative level of ANXA1 protein expression compared with SHAM group detected by immunohistochemistry is displayed. (D) Western blotting analysis of ANXA1 protein expression in PASMCs 24 h after different concentrations of ET-1 treated. β-actin was used as an internal control. All datas were representative of four independent experiments. Values were expressed as means±SEM. *p< 0.05 vs. control group. ^#^P< 0.05 vs. ET-1 treated group.

Simultaneously, immunohistochemical analysis showed that ANXA1 protein was highly expressed in pulmonary artery in sham lung, while it was much less expressed in HPS lung (Fig 2A, 2B and 2C). It indicates the reverse correlation between ET-1 and ANXA1. Thus, we studied the relationship between ET-1 and ANXA1 in PASMCs *in vitro*, Western blot demonstrated that treatment with ET-1 in a dose range of 1 nM-50nM for 24 h significantly decreased the ANXA1 expression in a dose-dependent manner (Fig 2D). This result indicates that ET-1 is an important negative regulator of ANXA1.

### ANXA1 is successfully expressed in PASMCs

**Fig 3:**
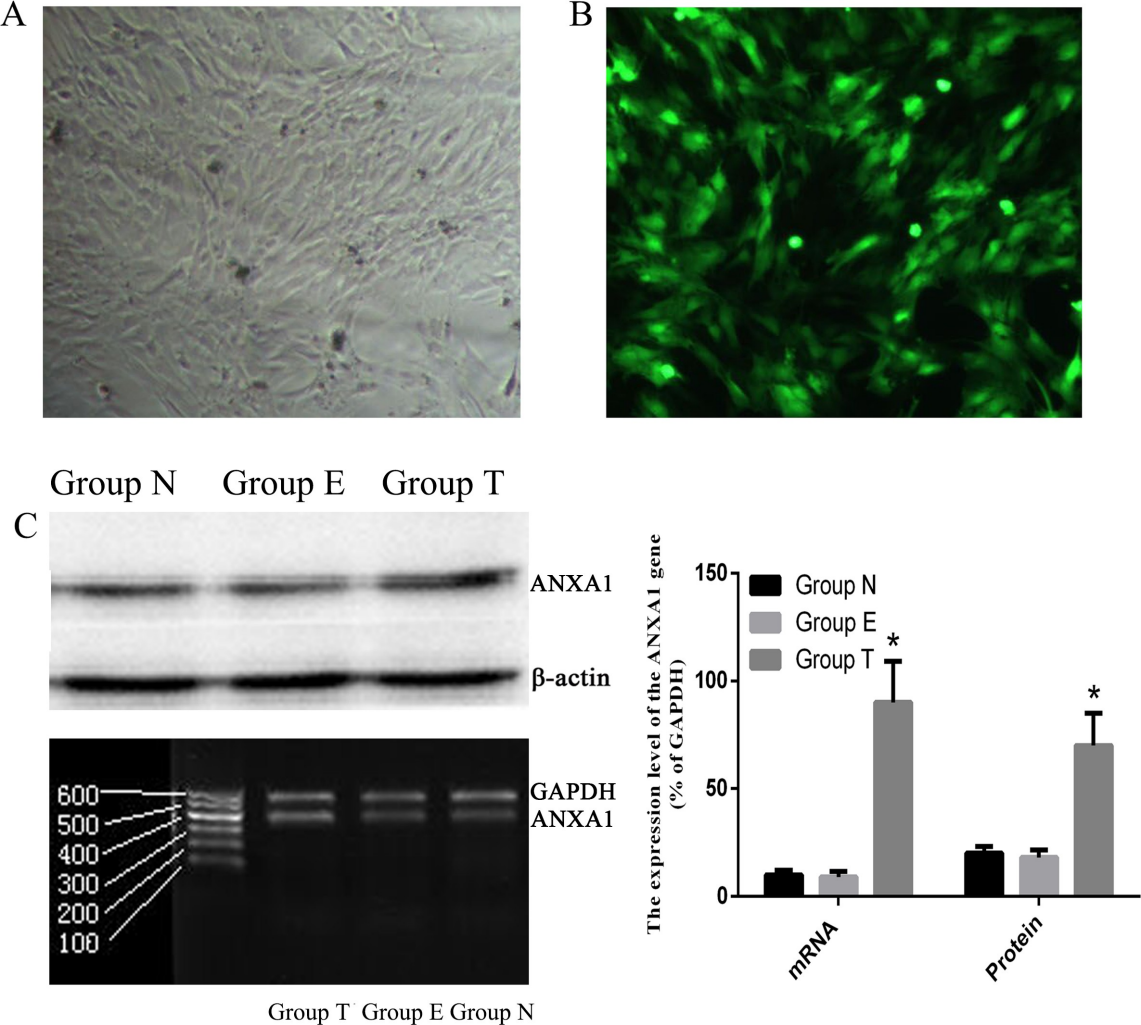
Ad-ANXA1 transfected into PASMCs was strongly expressed and the protein was found in both the cytoplasm and nuclei. (A) Morphology of PASMCs 24 h after transfection with Ad-ANXA1(magnification 100×). (B) Bright green fluorescence was observed in nearly all cells using an inverted fluorescence microscope following transfection with Ad-ANXA1 containing the green fluorescent protein (GFP) gene (magnification 100×). (C) The expression of the ANXA1 mRNA and protein in PASMCs 24 h after either the mock transfection or transfection with Ad-ANXA1 was determined by RT-PCR and western blotting analysis. GAPDH was used as an internal control in RT-PCR analysis and β-actin was used as an internal control in the Western blot analysis. Each data point represents the mean±S.E.M. of four independent experiments. *p<0.05 vs Group N.; Group N: Non-transfected group; Group T: transfected group; Group E: empty vector group.

To explore the effect of ANXA1 on PASMCs, we firstly tested the efficiency of ANXA1 ectopic expression in PSAMCs by transfection with Ad-ANXA1. PASMCs appeared to have a normal morphology and didn’t show any pathological characteristics following transfection with a 60-MOI-dose of Ad-ANXA1 for 24 h (Fig 3A). Meanwhile, ectopic expression of ANXA1 was detected in the cytoplasm and nuclei of nearly all cells (Fig 3B). At 24 h post-transfection with Ad-ANXA1, a significant increase in both transcript and protein levels of ANXA1 was observed (Fig 3C). These findings demonstrated that ectopic ANXA1 was efficiently expressed in PASMCs.

### ANXA1 overexpression inhibits the ET-1-induced inflammatory phenotype of PASMCs

**Fig 4:**
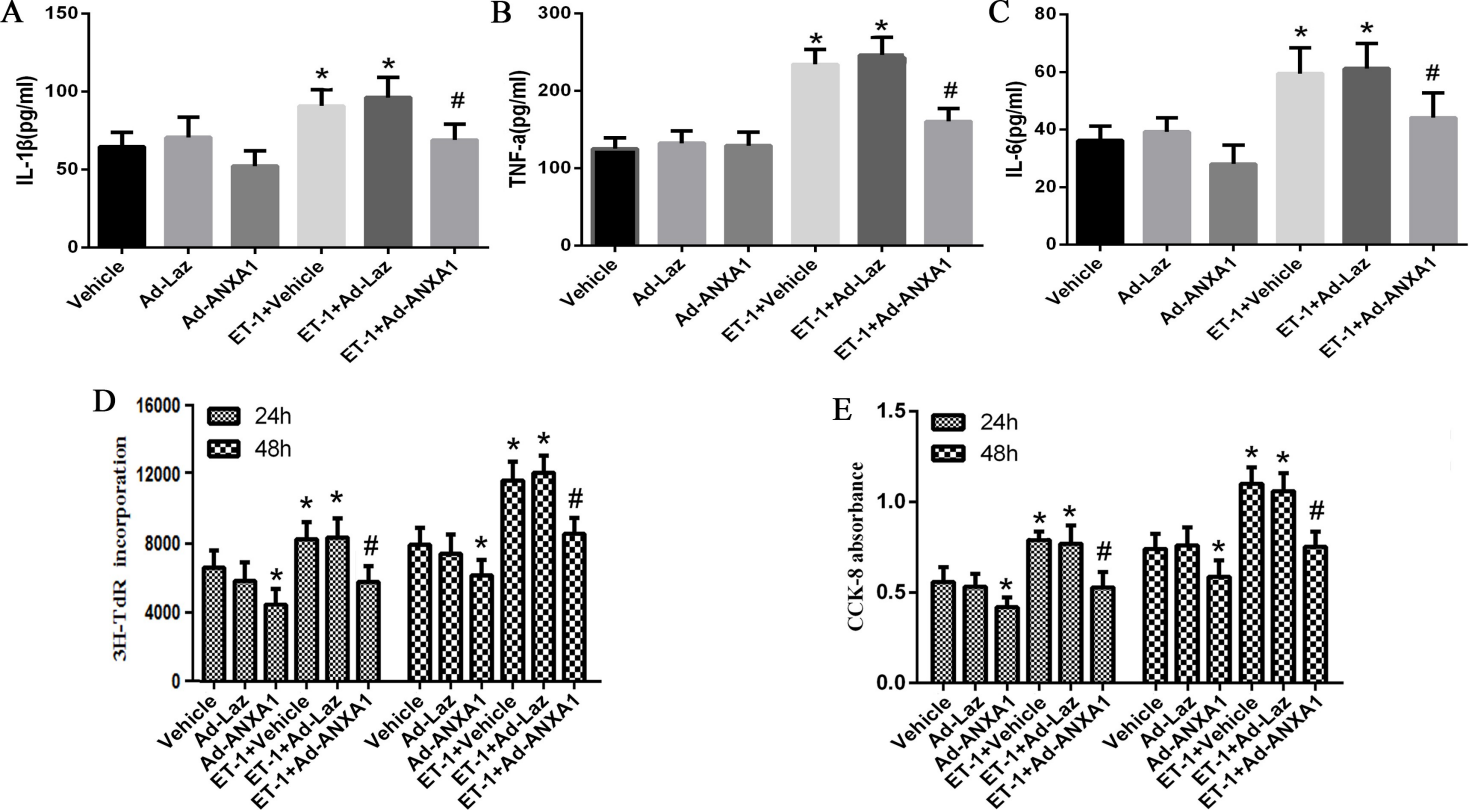
ANXA1 overexpression inhibited ET-1-induced inflammatory phenotype and proliferation of PASMCs. (A.B.C) PASMCs were treated with up to 50 nM of ET-1. Expression of IL-1β, IL-6 and TNF-a was measured in the supernatants of PASMCs by ELISAs at 24 h. (D.E) The change in the value of the incorporation of ^3^H-TdR and the absorbance of CCK-8 assay was displayed. The inhibitory effect was investigated at 24 h and 48 h. Each data point represents the mean ± S.E.M. of four independent experiments.*p< 0.05 vs. control group. ^#^P< 0.05 vs. ET-1 treated group.

Previous study has shown that inflammation play an important role in HPS induced PVR[17]. ET-1 is a pro-inflammatory effector while ANXA1 is an anti-inflammatory protein. Thus, we investigated the effects of ET-1 and ANXA1 on inflammatory phenotype in PASMCs. We treated PASMCs with 10 nM of ET-1, and then we measured the levels of IL-1, IL-6 and TNF-a in the supernatants of PASMCs by ELISA. The results displayed that the un-stimulated PASMCs exhibited low and basal release of IL-1β, IL-6 and TNF-a. However, the release levels of IL-1β, IL-6 and TNF-a in the supernatant of PASMCs were significantly increased under ET-1 stimulation. Interestingly and importantly, ectopic expression of ANXA by Ad-ANNXA1 transfection significantly impeded the upregulation of IL-1β, IL-6 and TNF-a levels (Fig 4A, 4B and 4C). These data demonstrated that ANXA1 inhibited the ET-1-induced inflammatory phenotype of PASMCs.

### ANXA1 overexpression inhibits the ET-1-induced proliferation of PASMCs

Next, we assessed the effect of ET-1 and ANXA1 in cell proliferation of PASMCs because PASMC proliferation has been reported to be involved in PVR. ^3^H-TdR incorporation assay showed that cell proliferation was significantly increased in the ET-1 treated group at each time point when compared to control group. However, with ectopic expression of pEGFP-ANXA1, cell proliferation was significantly inhibited in both control and ET-1 treated group (Fig 4D). Similar results were obtained from the CCK-8 viability assay (Fig 4E). These results confirmed a previously reported finding that ET-1 and ANXA1 promoted and repressed PASMCs proliferation, respectively [15].

### ANXA1 decreases both nuclear ERK1/2 accumulation and cyclin D1 expression in PASMCs

**Fig 5:**
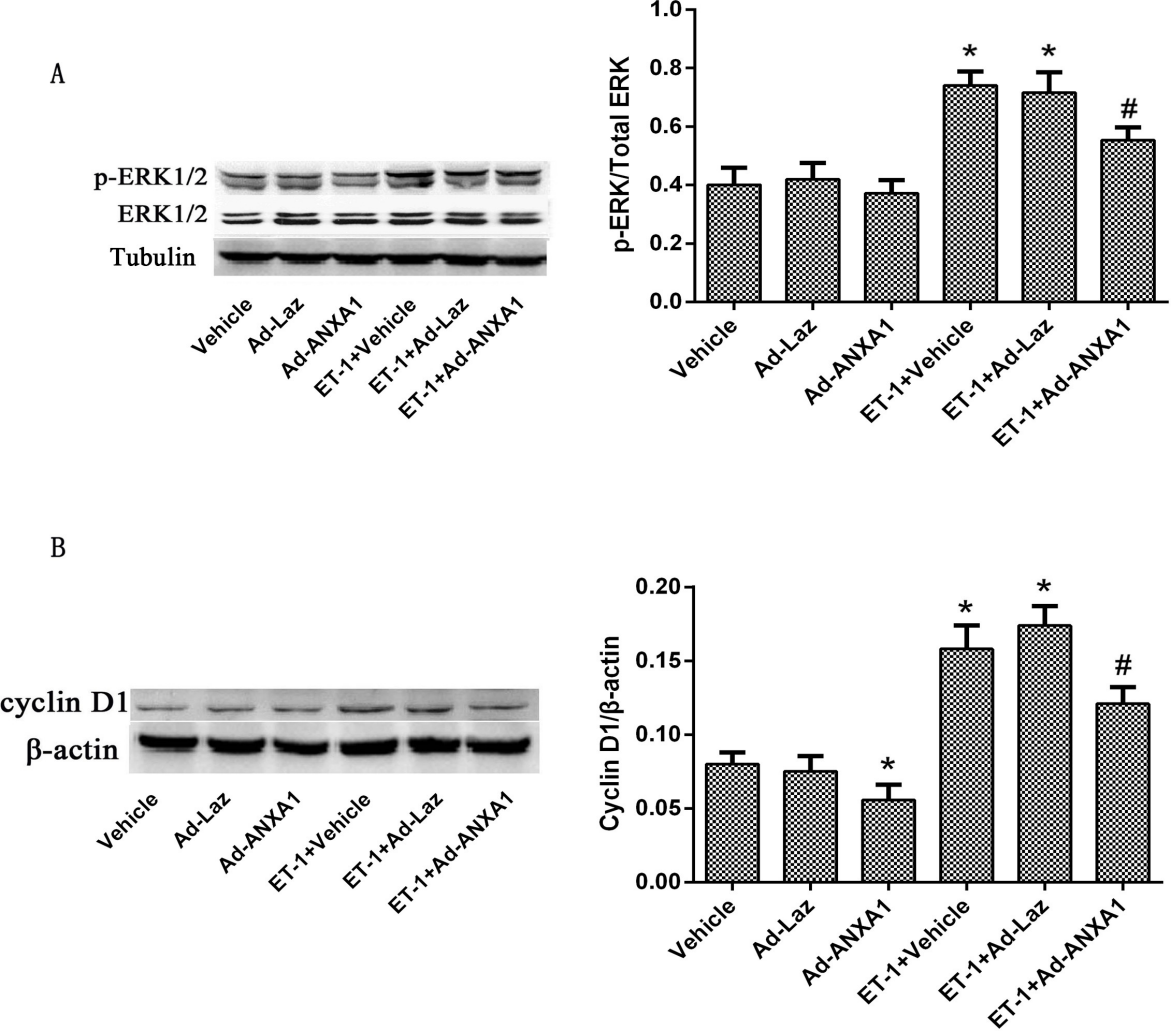
ANXA1 decreased p-ERK1/2 accumulation in the nucleus and decreased cyclin D1 expression in PASMCs. (A)PASMCs were transfected with Ad-ANXA1 48 h before ET-1-stimulation. Western blot analysis was used to measure the protein expression of total ERK1/2 and nuclear p-ERK1/2 expression in PASMCs 24 h after ET-1 treatment. (B) PASMCs were transfected with Ad-ANXA1 48 h before ET-1-stimulation. Western blot analysis was used to measure the protein expression of cyclinD1 expression in PASMCs 24 h after ET-1 treatment. β-actin was used as an internal control. Each data point represents the mean ± S.E.M. of four independent experiments. *p< 0.05 vs. control group. ^#^P< 0.05 vs. ET-1 treated group.

Next, we investigated how ET-1 and ANXA1 regulate inflammation and proliferation of PASMCs. As previous studies indicated ERK1/2 signaling pathway and cyclin D1 as main downstream factors of ANXA1,we determined both factors in PASMCs. Our western blot analysis showed that ET-1 stimulation had no effect on total ERK1/2, but increased the level of p-ERK1/2 in the nucleus when compared to control group. Consistent with the inflammation and proliferation phenotypes, the level of p-ERK1/2 in the nucleus was decreased by ANXA1 overexpression in both ET-1 stimulated group and control group (Fig 5A). Simultaneously, ET-1 significantly increased the cyclin D1 expression, while ANXA1 significantly inhibited the ET-1-induced increase in cyclin D1 expression in both ET-1 stimulated group and control group (Fig 5B). Thus, the respective changes of active ERK1/2 signaling pathway and cyclin D1 explained the controls of ET-1 and ANXA1 in PASMC inflammation and proliferation.

### ET-1 downregulates ANXA1 by posttranslational mechanisms

**Fig 6:**
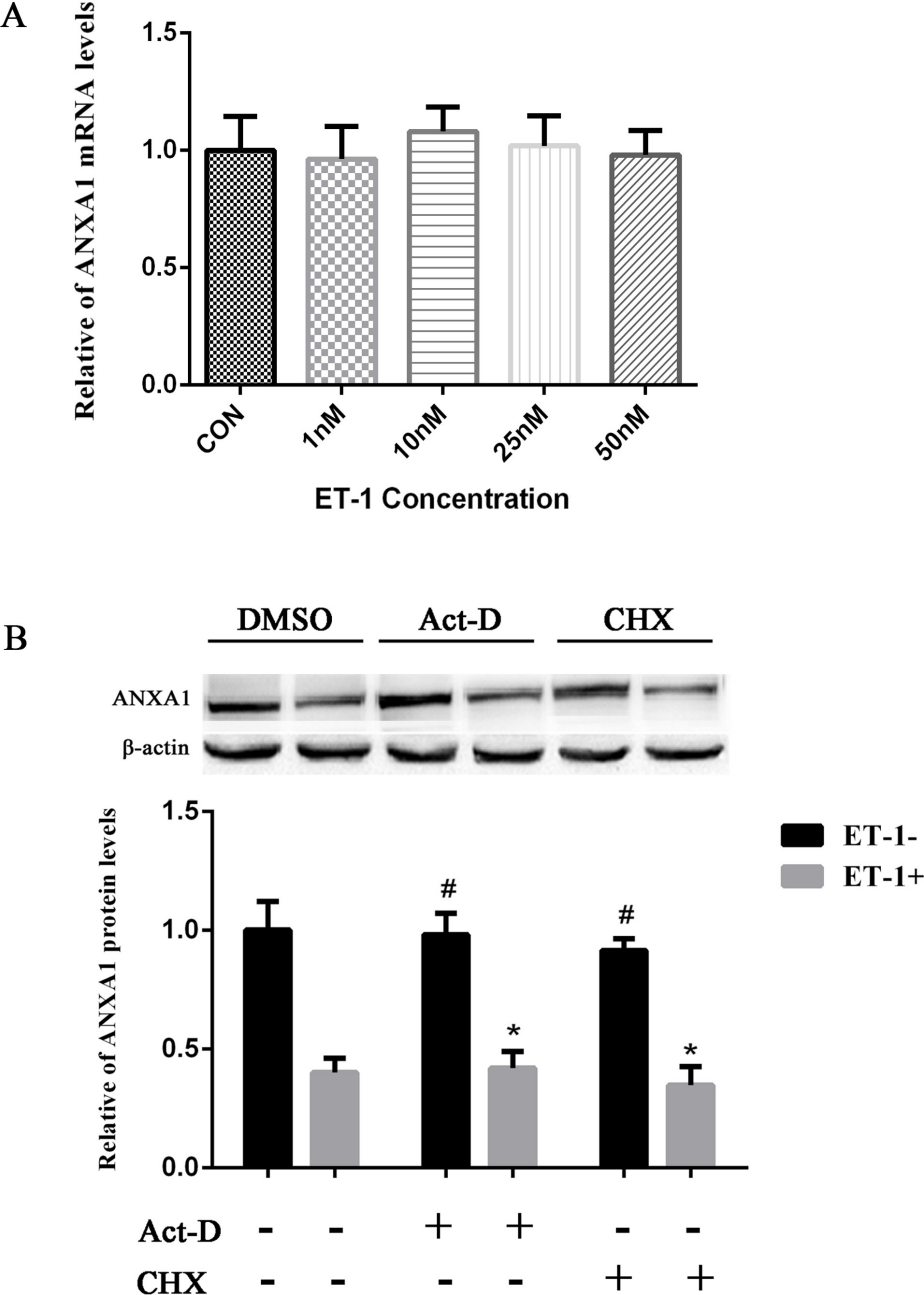
ET-1 down regulated ANXA1 by posttranslational mechanisms. (A)PASMCs were treated with different concentrations of ET-1 for 24 h, and then cells were harvested for ANXA1 mRNA level analysis by qPCR. Data were normalized to GADPH (P > 0.05, n = 5). (B)PASMCs cultures were pre-incubated with Act-D (2.5 μg/mL) or CHX (10 μg/mL) for 1 h, then treated with 10 nM ET-1 for an additional 24 h and ANXA1 protein was analyzed by using Western blot. Each data point represents the mean ± S.E.M. of four independent experiments.*p< 0.05 vs. ET-1 untreated control group. ^#^P< 0.05 vs. ET-1 treated control group.

Our following main question is how ET-1 negatively regulates ANXA1 protein level. To explore the regulatory mechanism of ET-1-mediated ANXA1 protein inhibition, we assessed whether ET-1 treatment can alter the ANXA1 mRNA expression in PASMCs using qPCR. As shown in Fig 6A, ET-1 treatments with different concentrations did not increase the ANXA1 mRNA levels when compared with control, indicating that ANXA1 was negatively regulated by ET-1 through a post-transcriptional mechanism. The next question is which post-transcriptional regulation is involved in this control. We firstly assessed the protein translation level of ANXA1 affected by ET-1. To do that, PASMCs were pretreated with translational inhibitor CHX (10 μg/mL), compared with transcriptional inhibitor Act-D (2.5 μg/mL) for 1 h followed by 24 h of treatment with 10 nM ET-1; and then the total proteins were collected and analyzed by using Western blot. As shown in Fig 6B, the reduced levels of ANXA1 by ET-1 were very similar in cells treated with or without Act-D or CHX, indicating that ET-1 does not influence the transcription and translation of ANXA1. All these results suggested that ET-1-mediated reduction of ANXA1 protein level may be due to the alteration of ANXA1 protein stability.

### Carbonylation of ANXA1 induced by the ET-1-mediated ROS decreases ANXA1 protein stability

**Fig 7:**
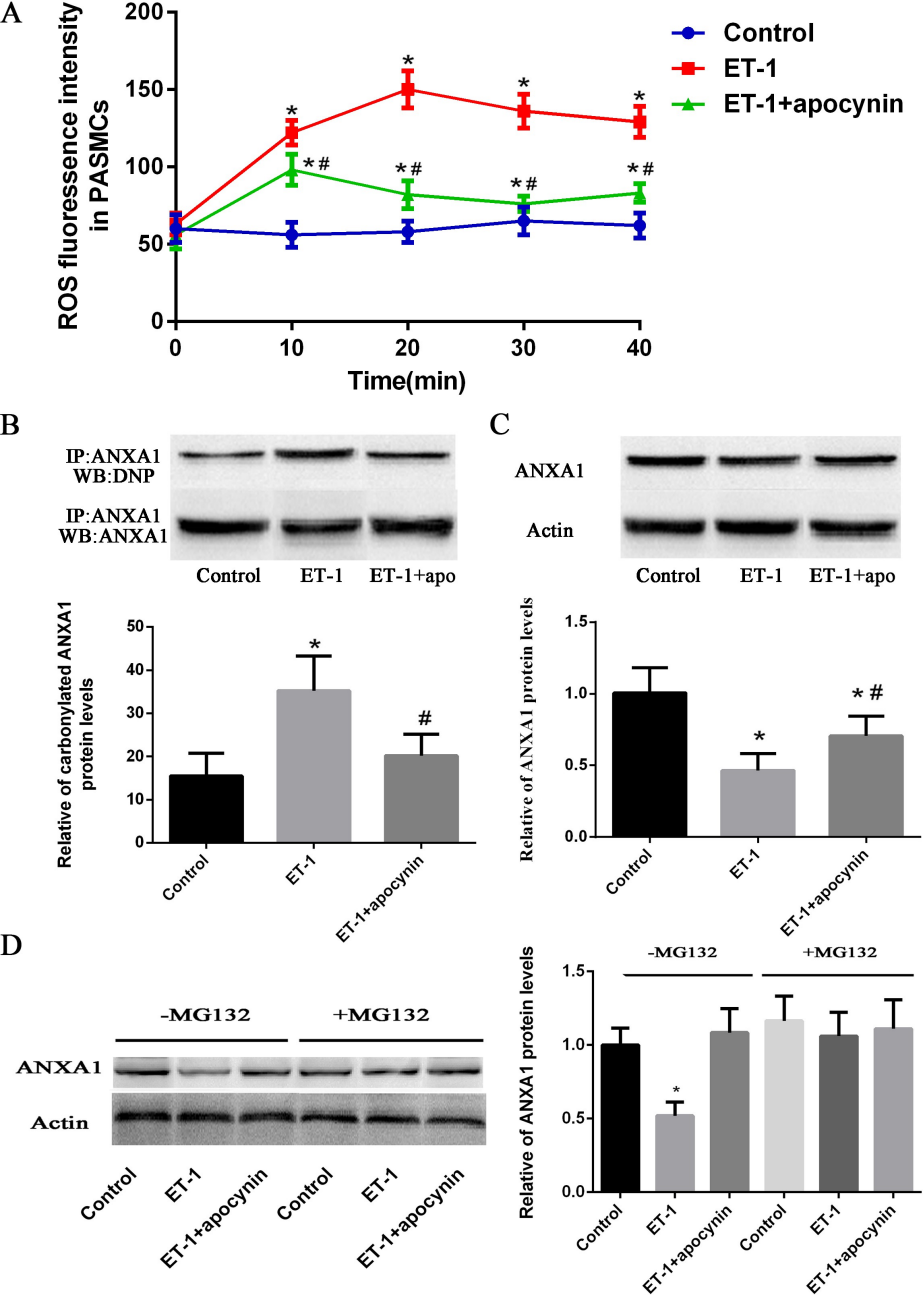
Carbonylation of ANXA1 induced by the ET-1-mediated ROS decreases ANXA1 protein stability. (A) PASMCs were treated with ET-1 (10 nM) in the presence or absence of Apo (100 μmol/l)for 60 min. PASMCs were stained by dihydroethidium (DHE), DHE fluorescence intensity was measured by flowcytometry analysis and expressed as fold induction compared with control group. (B) Cells were treated with 10 nM of ET-1 for 6 h, and whole cell extracts were immunoprecipitated with an anti-ANXA1 antibody. ANXA1 carbonylation was detected by Western blot using an anti-DNP antibody. (C) Whole cell extracts were assayed by Western blot analysis to measure ANXA1 levels. (D) PASMCs were pre-treated with MG132 (10 μM) for 1 h and then exposed to the ET-1 for 24 h; Western blot analysis to measure ANXA1 levels. Each data point represents the means ±SEMs for parametric tests; n = 4. *p< 0.05 vs. control group. ^#^P< 0.05 vs. ET-1 treated group.

As ET-1 has been known to generate intracellular ROS[18–20], we examined the ROS generation in PASMCs in response to ET-1. Intracellular release of ROS from PASMCs was measured by flowcytometry using DCFH-DA, in PASMCs treated for 40 min with a single dose of ET-1(10 nM). Compared with untreated PASMCs, treatment with ET-1 caused a significant increase in intracellular ROS generation at all time points. Pre-treatment of PASMCs with the NAD(P)H oxidase(the main source of ET-1-induced cellular ROS) inhibitor apocynin (100 μmol/l) for 30 min strikingly suppressed the ET-1-induced intracellular ROS generation (Fig 7A).

As Suzuki and co-workers have previously reported that ROS can carbonylate and degrade ANXA1[15], we checked the carbonylation level of ANXA1 by immuno-precipitation and western blot analysis. Cells were treated with 10 nM ET-1 for 6 h, whole cell extracts were immunoprecipitated with anti-ANXA1 antibody, and then carbonylated ANXA1 was detected by immunoblotting. By densitometry, Carbonylated ANXA1 was 2.7 times higher in the ET-1-treated cells than in controls (Fig 7B). Furthermore, ANXA1 levels were 1.9 times lower in the ET-1-treated PASMCs than in controls (Fig 7C). Next, we checked the inhibitory effect of protein degradation. To do that, PASMCs were pre-treated with a proteasome inhibitor, MG132 (10 μM) for 1 h followed by 24 h of treatment with 10 nM ET-1, and then the total proteins were collected and analyzed by using Western blot. Compared to MG132 untreated Group, the decreased protein levels of ANXA1 induced by ET-1 were significantly increased in MG132 pre-treated Group (Fig 7D). These data suggested that carbonylation of ANXA1 resulted in the proteasome-dependent ANXA1 degradation.

## Discussion

Liver cirrhosis and other chronic liver dysfunction can lead to reduced hepatic clearance or enhanced production of circulating mediators, including growth factors and cytokines, both of which are the major contributors to PVR. However, the mechanisms controlling PVR in HPS are poorly understood. Our previous study indicated that inflammation and proliferation of PASMCs may play an important role in this process[21]. Unlike skeletal and cardiac muscle cells, where cellular differentiation is coupled to an irreversible exit from the cell cycle, PASMCs maintain the ability to proliferate after terminal differentiation[22]. Stimulations such as hypoxia, various growth factors and mechanical stretch usually induce quiescent PASMCs to an inflammatory phenotype and re-enter the cell cycle[23]. For a long time, phenotypic modulation and proliferation of PASMCs has been the main focus of studies in PVR in HPS and other pulmonary vascular disease. Previous studies and our initial research have demonstrated that HPS serum induces the abnormal phenotypic modulation and proliferation of PASMCs. But the molecular underlying mechanisms involved in this event in HPS remain elusive.

It is well established that ET-1 contributes to the pathological remodeling of vasculature in a number of diseases including hypertension, diabetes and restenosis[24, 25]. The binding of ET-1 with ET-A receptors on vascular smooth muscle cells promotes vasoconstriction and cell proliferation[26]. In the current study, ET-1 staining was highly observed in PASMCs, alveolar epithelial cells and macrophages in HPS rat, while ANXA1 staining was decreased in PASMCs. This implicated the reverse correlation between these two factors. Consistent with this hypothesis, we demonstrated that ET-1 containing media decreased the ANXA1 expression in PASMCs in a dose-dependent manner.

Our current study demonstrated ANXA1 as the main downstream effector of ET-1 mediated PVR in HPS. ANXA1 has been mainly studied in immune response regulation, as a mediator of the effects of glucocorticoids through the inhibition of PLA2 activity, ANXA1 has been discovered to regulate cyclooxygenase and inducible nitric oxide synthase expression, and subsequently its serves as a main regulator of proliferation, differentiation and apoptosis[27, 28]. A series studies have shown that downregulation of ANXA1 expression is associated with the increased cell proliferation. Previous work from in vitro cultured cells suggested that ANXA1 can inhibit the growth and proliferation of A549 lung cancer cells and prostate cancer cell lines[29, 30]. ANXA1 also has anti-proliferative activity in macrophages due to the constitutive activation of the MAPK/ERK pathway, which was linked to its phosphorylation by epidermal growth factor[31]. However, the detailed molecular mechanisms have not been defined in PASMCs. In this study, we showed that ANXA1 protein expression was decreased in HPS model. Re-expression of ANXA1 inhibited the ET-1 induced nuclear p-ERK accumulation and cyclin D1 expression, and thus it caused subsequent inhibition of PASMCs phenotypic modulation and proliferation.

Next, we identified carbonylation as the main post-translational control of ANXA1 mediated by ET-1. Protein carbonylation and decarbonylation have been reported as possible mechanisms for ROS signaling-mediated protein stability[15, 32–34]. Excessive ROS often cause certain amino acids such as Arginine, Lysine, Threonine, and Proline to be carbonylated under oxidative stress[35–37]. Some of carbonylated amino acid residues at specific positions in a protein polypeptide chain may result in significant changes in the function of target proteins. Protein carbonylation has been shown to cause reduced ligand binding properties, reduced enzymatic activities, increased susceptibility to proteolytic activities[38, 39]. In this study, we confirmed the findings by Suzuki and co-workers[15] that ET-1 induced ROS production can decrease the ANXA1 protein stability through the carbonylation—mediated proteasome pathway.

To summarize, our data demonstrated the molecular regulatory mechanism of ANXA1 by ET-1 that was previously described by Suzuki and co-workers[15] also regulates PVR of HPS. Increased ET-1 levels induced the carbonylation and degradation of ANXA1, and thus the decreased ANXA1 led to the PASMCs proliferation, possibly by inhibition of nuclear p-ERK accumulation and cyclin D1 expression. These results suggest that upregulation of ANXA1 protein expression and stability could be a potential therapeutic strategy to modulate PASMCs functions, which impede PVR and HPS progression.

## Supporting information

S1 File The S1_File.xls file contains minimal data of this study. This file contains seven separate parts. Seven parts correspond to the seven results in this study. In case of any questions or inquiries please contact: lukaizhi2010@163.com.
(XLSX)
